# The rise of promotional rhetoric in the Discussion Sections of an interdisciplinary field: a diachronic perspective

**DOI:** 10.3389/fpsyg.2025.1685487

**Published:** 2025-12-03

**Authors:** Yumei Zhu, Xuemei Li

**Affiliations:** School of Foreign Languages, Sichuan University of Arts and Science, Dazhou, China

**Keywords:** promotional step, hyping stance marker, diachronic change, interdisciplinary field, research evaluation

## Abstract

In recent decades, the growing competition to publish internationally has made the Discussion section increasingly promotional, as authors interpret and evaluate their findings to highlight research significance. However, this section also risks exaggerating a study’s contribution with promotional discourse. The current study examines the diachronic changes of promotional strategies in Communication research articles (RAs) Discussion sections by analyzing two corpora from 1980 to 1981 and 2020–2021. Drawing on analytical frameworks for analyzing promotional steps and stance features, findings revealed a marked increase in the frequency of promotional steps and stance markers. The observed changes indicated a shift toward more implicit, subtle and strategical forms of persuasion. This study contributes to the study of promotional discourse in academic writing and calls for greater recognition of rhetorically modest and transparent writing practices as markers of scholarly credibility, encouraging editorial and review policies that reward integrity alongside visibility.

## 1 Introduction

In today’s increasingly competitive academic landscape, shaped by a pervasive ‘publish or perish’ culture, researchers are under mounting pressure to secure recognition, funding, and career advancement through publication (Hyland, 2023). In this intensified climate, scholarly writing has evolved beyond the mere dissemination of knowledge; academics are expected not only report their findings but also assert the research innovation, significance and originality. This dimension of academic writing, often described as promotional discourse, has become a subject of growing debate. On the one hand, promotional discourse is viewed as a necessary rhetorical tool for convincing reviewers, editors, and the broader disciplinary community of the merit of their work (Cheng, 2021). On the other hand, it is often criticized for encouraging overstated or exaggerated claims that may misrepresent results, inflate the perceived significance of research, and ultimately erode public trust in scientific communication (Millar et al., 2019; Boutron and Ravaud, 2018). In response, scholars increasingly call for a balanced approach that considers the communicative needs of both writers and readers (Li et al., 2025). Yet, concrete guidance on how such a balance is rhetorically achieved remains scarce, particularly in the Discussion section.

Although promotional discourse has been widely examined, particularly through diachronic studies that chart its rise over time (Cao et al., 2021; Wen and Lei, 2022; Wu and Pan, 2023), most research has focused narrowly on the Abstract section (Vinkers et al., 2015; Edlinger et al., 2023; Liu and Zhu, 2023). Yet Abstracts, by design, are brief and highly conventionalized, offering only limited scope for authors to negotiate stance, contribution, and evaluative claims. This narrow focus provides an incomplete picture of the broader rhetorical mechanisms through which authors promote their work within the article. By contrast, the Discussion section performs distinctive communicative purposes and is inherently promotional, as it goes beyond merely presenting results by interpreting findings, evaluating contributions, and projecting limitations and implications for future research. It displays unique patterns of promotion and linguistic choices compared to other sections (Yang and Allison, 2003; Liu and Buckingham, 2018; Millar et al., 2019). Despite this, the diachronic development of promotional discourse in Discussion sections remains underexplored, leaving a critical gap in our understanding of how persuasion is constructed in the closing stages of academic argumentation.

In addition, most existing studies often solely focused on isolated linguistic devices, such as attitude marker, booster and hedge, but without considering how these choices related to underlying rhetorical purposes. This fragmentating risks obscuring the interaction between rhetorical moves and stance markers, which together form the scaffolding of persuasive academic discourse. For example, increases in positive evaluative language are often attributed to heightened competition (Cao et al., 2021; Vinkers et al., 2015), but such explanations are overly general and fail to account for how specific communicative goals drive these linguistic patterns.

A third and equally important gap concerns the role of interdisciplinarity in shaping promotional discourse. As academic research increasingly crosses disciplinary boundaries, fields such as Communication, which draws on methods and theories from sociology, psychology, linguistics, and media studies (Craig, 1999) provide a compelling site for investigating how disciplinary hybridity affects rhetorical strategies. Yet, to date, little is known about how interdisciplinary dynamics influence the evolution of promotional discourse, especially in the Discussion section.

To address these gaps, the present study investigates the diachronic development of promotional discourse in the Discussion sections of Communication RAs, focusing on the interaction between promotional steps and stance markers. Drawing on an adapted framework of promotional strategies (Moreno, 2021) and Hyland’s (2005) stance model, we analyze two corpora of Discussion sections published in 1980–1981 and 2020–2021 to examine how academic authors construct persuasion across time.

This study makes three key contributions to the field. First, it redirects scholarly attention from the overexamined Abstract to the underexplored Discussion section, offering insights into how promotional discourse functions where authors consolidate claims and project future research. Second, it introduces a dual-layered analytical framework that integrates rhetorical moves with stance markers, providing a more functionally grounded and holistic view of persuasion in academic writing. Third, by focusing on Communication, a hybrid field bridging empirical and interpretive traditions, this study examines how methodological hybridity makes persuasion rhetorically demanding. Communication scholars must balance empirical rigor with reflexive interpretation, negotiating credibility across diverse evaluative norms. This disciplinary setting thus provides a fertile ground for exploring how hybridity shapes evolving conventions of academic promotion. Together, these contributions enrich our understanding of genre evolution and offer practical implications for research evaluation.

## 2 Literature review

### 2.1 Promotional rhetoric in Discussion section

Previous studies have examined various aspects of Discussion sections, including their rhetorical structure in Dentistry (Basturkmen, 2012) and Applied Linguistics (Liu and Buckingham, 2018; Ash’ari et al., 2023), argumentative patterns (Parkinson, 2011), phraseological features in specific rhetorical moves (Le and Harrington, 2015; Montgomery, 2023), the use of interpersonal resources in representing and negotiating conflict (Cheng and Unsworth, 2016), and the forms and functions of negation in discussing limitation of PhD theses (Sun and Crosthwaite, 2022). A common feature identified in these studies is that the Discussion section is interpretative, extending beyond the factual reporting of results to include the comparison and evaluation of findings within broader research and social contexts (Yang and Allison, 2003; Basturkmen, 2012; Moreno and Swales, 2018). From a genre-based perspective, this interpretative stance often entails a promotional dimension, whereby authors emphasize the significance of their study, articulate how it addresses existing research gaps, demonstrate its practical relevance, and frame its limitations as opportunities for future inquiry (Moreno and Swales, 2018; Montgomery, 2023).

The promotion dimension in the Discussion section has been categorized into direct and indirect steps (Lindeberg, 2004). Direct promotional steps include centrality claims, statements of research gaps, and the use of boosters, all of which explicitly highlight the significance of the study. In contrast, indirect promotional strategies encompass caveats and limitations. As Lindeberg (2004) explains, these indirect elements may initially appear to diminish the perceived value of the research—by acknowledging weaknesses (caveats and limitations) or by challenging existing work (counterclaims and recommendations). However, their use ultimately enhances the credibility and legitimacy of the study by demonstrating transparency and scholarly engagement, thereby contributing to a more nuanced and persuasive rhetorical stance (Lindeberg, 2004). Further, based on this categorization, Moreno (2021) further subdivided boosters into three promotional steps: contribution of the current study, positive features of the current study, and applicability of results or usability of outcomes to identify, and add hypothesizing for future research into the indirect promotional steps (Table 1).

**Table 1:** Direct and indirect promotional steps by Lindeberg (2004) and Moreno (2021).

Category	Lindeberg (2004)	Moreno (2021)
Direct	Claims of Centrality	
	Gaps	Gap or deficiency in others’ research or practice, or problem
	Boosts	Applicability or usability of outcomes
		Contribution of the current study
		Relevance of topic and/or positive evaluation of the state of knowledge or practice.
Indirect	Caveat	
	Limitation	Pointing out negative features or limitations of the current study
	Counterclaim	
	Future research	Making recommendations for future research or practice
		Hypothesizing for future research

Another body of research has examined cross-cultural differences in promotional strategies, with a general consensus that English RA Discussion sections exhibit a higher degree of overt promotional rhetoric compared to those written in other languages (Moreno, 2021; Deng et al., 2024; Loi et al., 2016). For instance, the most notable difference between English and Spanish social science RAs lies in their use of direct promotional strategies. Spanish authors tended to emphasize the contribution of their current study, whereas English authors were more inclined to highlight the positive features of the current or proposed research (Moreno, 2021). Moreno attributed these differences to the traditions of the Spanish research community and the broader cultural norm of modesty. In the comparative study between the Chinese and English Discussion section of Applied Linguistics, English writers were found to highlight the value of their research while acknowledging its limitations, whereas Chinese writers tended to adopt a direct promotional strategy by addressing the research gaps identified in the Introduction (Deng et al., 2024). They also attribute these differences to the writer–reader roles in academic writing culture and the influence of traditional Chinese cultural values of humility.

While these studies illuminate how socio-cultural conventions mediate persuasive practices across languages, they remain largely intercultural in scope. Over the past few decades, EAP instruction and the intensification of international academic exchange have blurred many cross-cultural differences in rhetorical practices. For example, diachronic studies have shown that English RAs by Chinese authors increasingly adopt rhetorical strategies typical of international Introductions in Applied Linguistics (Yin et al., 2023). While disciplinary conventions such as research paradigms, methodological preferences, and levels of interdisciplinarity, may play an even greater role in shaping rhetorical practices. For example, in emphasizing research centrality, Anthropology Introductions tend to highlight real-world relevance, whereas Psychology and Sociology foreground centrality by engaging in scholarly debates (Lu et al., 2021). Yet, how such diachronic and disciplinary development have influenced promotional strategies in Communication Discussion sections remains largely underexplored.

### 2.2 Stance markers as hyping discourse

Stance in academic writing refers to the ways writers express their attitudes, judgments, and commitment toward the propositions they present (Biber, 2006). It reflects the writer’s evaluation of knowledge claims, positioning themselves in relation to the disciplinary community and ongoing scholarly conversations (Hyland and Jiang, 2016). Within the study of stance, there is a division between attitudinal and epistemic perspectives. An attitudinal stance conveys the writer’s affective or evaluative orientation, such as expressing emotions or value judgments through discourse markers, for example, “fortunately” or “interestingly” (Ochs and Schieffelin, 1989). On the other hand, an epistemic stance is concerned with the degree of certainty, reliability, or limitation a writer attaches to a proposition, signaling how firmly a claim is held or how precisely it is stated, for example, “may” and “possibly” (Hyland and Jiang, 2016).

Hyping discourse that aims to enhance the perceived value of research has been examined through various rhetorical features, including the use of positive and negative words (Cepeda et al., 2015; Bordignon et al., 2021), hyperbolic and/or subjective language to exaggerate findings (Millar et al., 2019, 2020), boosters and positive attitude markers (Hyland and Jiang, 2021, 2024), as well as hedges that help mitigate research uncertainties (Yao et al., 2023). Although explored from different perspectives, the hyping discourse described above can be understood through the stance framework, which captures how writers use boosters and attitude markers to emphasize significance, while employing hedges to manage uncertainty and maintain academic credibility. It is noteworthy that in the series of studies by Hyland and Jiang (2021, 2024), boosters and positive attitude markers are included as hyping elements. However, negative attitude markers such as the word “crisis” can also serve a hyping function by drawing attention through overt statements of potential risks, uncertainties, or unknowns associated with the research (Weingart, 2017).

It is important to clarify that promotion and hype have been used interchangeably in some studies. In the present study, promotion is conceptualized as a broader rhetorical goal aimed at enhancing the perceived value of research, whereas hyping denotes its lexical and evaluative realization through amplifying devices such as boosters, hedges, and attitude markers.^1^

### 2.3 Diachronic changes of promotional rhetoric and hyping stance markers

To date, there has been limited diachronic investigation into the evolution of promotions in the Discussion section. One relevant study was the study by Martín and León Pérez (2024), which analyzed the Abstracts of medical RAs from 1940 to 2020. Their findings revealed an increasing presence of promotional steps over time, particularly the step of “stating the significance or contribution of the research findings,” which bears direct relevance to the communicative purposes of the Discussion section. However, the predominance of Abstract-focused research has produced an imbalanced picture of diachronic change. Abstracts are short, formulaic, and highly standardized across journals, making them more accessible for large-scale corpus analysis and easier to compare diachronically. In contrast, Discussion sections are longer, rhetorically diverse, and often merged with Results or Conclusions (Lin and Evans, 2012), posing challenges for corpus retrieval and structural alignment. Ironically, this very complexity in which authors interpret findings, evaluate contributions, and project implications makes the Discussion section the most inherently promotional part of the RA. Yet its diachronic evolution remains underexplored.

Several diachronic studies have investigated the changing patterns of stance markers in the Discussion sections, revealing a consistent downward trend in their use within the heavily studied field of Applied Linguistics (Rezaei et al., 2021; Arizavi et al., 2023; Xie et al., 2024). This decline suggests a growing shift toward objectivity in academic writing conventions, with authors becoming increasingly cautious when expressing personal judgment, commitment, or affect, and making strategic use of stance markers. Such linguistic conventions allow writers to project epistemic credibility while minimizing overt self-promotion, thereby aligning with the broader scientistic ethos that privileges measurability, transparency, and evidential reasoning. While these findings offer valuable insights into some linguistic tendencies, the changing pattern of stance markers was observed in isolation, without taking into account how these stance markers functioned (often in combination) to achieve a certain communicative purpose.

Promotion, in particular, is not solely achieved through explicit language but also conveyed subtly and implicitly through a range of rhetorical devices (Cheng, 2021; Wang and Yang, 2015). For instance, the contribution statement “This investigation has the potential to enhance our understanding of…” includes the overtly promotional word “enhance,” which is mitigated by the hedge “potential.” Some diachronic studies have concluded that an increase in exaggerated or hyperbolic language signals intensified promotional discourse. However, such interpretations may be reductive if they overlook the mitigating role of hedging.

Given the limited diachronic research on the promotional discourse in the Discussion section of the interdisciplinary field-Communication, there is a clear need to investigate how these rhetorical features have evolved and interacted over time.

To address this gap, the present study aims to address the following research questions:

1. Have the frequencies of promotional steps in Communication Discussion sections changed diachronically?
2. What are the diachronic changes of stance markers across promotional steps?
3. How has the distribution of stance markers within promotional steps shifted over time, and to what extent do these changes reflect evolving rhetorical strategies?

## 3 Methodology

### 3.1 Corpus compilation

This is part of a larger study that carried out a diachronic investigation of the communicative function of the Discussion section in Communication RAs. The data in this study was sourced from four leading Communication journals, namely Communication Research (CR), Human Communication Research (HCR), Journal of Applied Communication Research (JOACR), and Communication Quarterly (CQ). According to the “Aim and Scope” section of the journal website, all four journals cover a wide variety of communication-relevant issues and particularly encourage methodologies, theories, and ideologies across a broad range of disciplines. To diachronically trace the changes in promotional strategies in the Discussion section, RAs from 1980 to 1981 and 2020–2021 were collected. In the two corpora, a random selection of five articles from each of the four journals in each year was employed to guarantee a total of 40 RAs in each period. Six JOACRs in the 1980 period were collected, as only four RA with separate Discussion sections was available in 1981, following other diachronic studies (Hyland and Jiang, 2018; Qin, 2004).

The corpus of 40 Discussion sections per period is sufficient for diachronic comparison. This size aligns with previous studies (e.g., Xie et al., 2024, 30 per period; Ash’ari et al., 2023, 20 RAs as the total corpus) and reflects the labor-intensive nature of qualitative move-step and stance annotation, for which small but balanced corpora are widely accepted (Guo and Lim, 2024; Yin et al., 2023). Moreover, Communication became an independent field only in the 1980s, when few specialized journals existed: Communication Studies was known as Central States Speech Journal until 1988, and the International Journal of Communication was launched only in 2007, so this sampling ensures both feasibility and representativeness.

Table 2 summarizes the basic information of the corpus.

**Table 2:** Basic corpus information.

No.	Journal	1980–1981		2020–2021	
		Articles	Number of words in discussion section	Articles	Number of words in discussion section
1	JOACR	10	5,052	10	13,693
2	HCR	10	8,473	10	15,710
3	CR	10	6,004	10	14,026
4	CQ	10	6,686	10	14,724
Total		40	26,205	40	58,153

We acknowledge that corpus comparability is a common challenge in diachronic genre research, as earlier datasets often contain fewer standalone Discussion sections. For example, in the diachronic study by Arizavi et al. (2023), only 21% of papers in the 1980–1990 corpus included a separate Discussion section, compared with 81% in the 2010–2019 corpus. In the present study, the difference was more moderate: 54% of the earlier articles and 71% of the later ones contained distinct Discussion sections, suggesting that the two sub-corpora are broadly comparable in structural composition (see Appendix A).

Another challenge concerns the size of the corpus, as can be seen in Table 2. To deak with the challenge, following previous diachronic studies (e.g., Li et al., 2025), the counts of each promotional step was based on the number of Discussion sections containing that step, rather than on the total frequency of its occurrence within those sections. The statistical tests of significance (Table 3) were also conducted on the counts of Discussion sections containing that step. Because both sub-corpora comprise the same number of Discussion sections, this measure ensures statistical comparability even though the earlier corpus contains fewer words overall.

**Table 3:** Changes promotional steps during two periods.

Move/step	Total N of CDs containing the steps				Total N of the steps in the corpus		
	1980–1981	2020–2021	*p*-value	chi-square	1980–1981	2020–2021	changes
					No.	No.	
Move 2: Reporting or restating key results							
Step 3: Highlighting results	17 (43%)	32 (80%)	0.000	11.850	27	61	126%
Move 4: Evaluating the current study or other research or practice							
Step 1: Pointing out the limitations of the current study	16 (40%)	30 (75%)	0.002	10.026	38	168	342%
Step 2: Stating the contribution or positive features of the current study	19 (48%)	35 (88%)	0.000	14.587	37	129	249%
Step 3: Noting specific gaps in other research or practice	11 (28%)	21 (53%)	0.022	5.208	11	34	209%
Move 5: Drawing implications							
Step 1: Suggesting the practical applicability of results	11 (28%)	27 (68%)	0.000	12.832	35	117	234%
Step 2: Making recommendations for future research	26 (65%)	36 (90%)	0.007	7.168	98	232	137%

### 3.2 Analysis framework

The data of this study was managed through promotional step annotation and the identification of stance markers. The promotional steps in our study are defined by the Swalesian genre analysis (1990), in which a step refers to text fragments that, individually or in combination, serve specific communicative purposes. The annotation of promotional steps was based on the promotional framework of the Discussion section by Moreno (2021), with small adjustments made, as the framework was initially designed for intercultural comparison (the refined promotional framework is shown in Table 4). The adjustments also referred to relevant studies in the Discussion sections by Lindeberg (2004) and Deng et al. (2024).

**Table 4:** Revised promotional framework of Discussion section.

Revised promotional moves and steps
Move 2: Reporting or restating key results
Step 3: Highlighting results
Move 4: Evaluating the current study or other research or practice
*Step 1: Pointing out the limitations of the current study*
Step 2: Stating the contribution or positive features of the current study
Step 3: Noting specific gaps in other research or practice
Move 5: Drawing implications
Step 1: Suggesting the practical applicability of results
*Step 2: Making recommendations for future research*

Note: The italicized steps are indirect promotional steps.

The stance markers in this study followed Hyland’s (2005) stance framework, where rhetorical choices convey personal assessments and feelings, including hedges, boosters, attitude markers, and self-mention. Although several previous studies have focused solely on boosters and positive attitude markers (see Hyland and Jiang, 2021, 2024) as hyping elements, our study also included hedges and negative attitude markers, which, as discussed in section 2.2, contribute to achieving the hyping effects. In previous studies, self-mention was included as part of the stance framework; however, this marker was excluded in the present study. Arguably, this discourse marker pertains to the writer’s presence and engagement with the audience, which potentially helps construct persuasive writing and publicize writers and their research (Harwood, 2005; Li, 2021). However, some functions of self-mention, such as discourse self, and autobiographical self (Ivanic, 1998), do not directly aim to elevate the propositional content or bolster the claims made within the research. To avoid any potential ambiguity, this study focused only on hedges, boosters, and attitude markers as the stance markers, named as hyping stance markers in this study.

### 3.3 Analytical procedure

The analytical procedure of this study consisted of two major processes. Firstly, the promotional steps were coded following the refined analytical framework of Moreno (2021) (see Table 4). The identification of steps was based on a refined method, that is, it must include new propositional meaning crucial for advancing the text toward the achievement of the communicative purposes (Moreno and Swales, 2018). In other words, the identified promotional step should contain at least one verb or in an elliptical form that can be easily converted into a verb from. This identification process was also assisted with linguistic clues, such as the p-phrase frame in limitation and recommendation steps (Montgomery, 2023), linguistic realization in claiming theoretical values in the Discussion section (Cheng, 2021), and the semantic meaning of the content.

After that, steps were grouped into broader functional categories (moves). To ensure coding consistency, all identified steps and hyping stance markers were re-coded by the same researcher after a one-month interval to allow cognitive distance and minimize recall bias, following Basturkmen (2012). Because Communication is a highly interdisciplinary field encompassing diverse subareas (e.g., political and health communication), employing an additional expert coder was impractical. To address this, the two rounds of coding were compared in MAXQDA 2020 (Coder 1 vs. Coder 2), yielding Cohen’s *κ* values of 0.832–0.953, indicating excellent reliability (Landis and Koch, 1977). Any remaining discrepancies were resolved through close rereading and consultation with specialist informants familiar with the relevant subfield. The results were recorded in the qualitative analysis software MAXQDA, and a Chi-square test was conducted to analyze the distribution of promotional steps across the two periods.

To quantify the use of hyping stance markers in the promotional steps across time, the categorization of attitude markers, boosters, and hedges was firstly done based on Hyland’s (2005) framework, supplemented by reference to the literature on relevant studies, such as hype discourse (Millar et al., 2019), positive words (Vinkers et al., 2015; Cao et al., 2021), appeals (Wang and Yang, 2015; Cheng, 2021), and metadiscourse markers in Discussion section (Rezaei et al., 2021; Liu and Buckingham, 2018; Hyland and Jiang, 2021). The following example illustrated typical examples of hyping stance markers in the corpus. Example 1 illustrates the step that *highlights the contribution of the study*. In this example, the single lined word “can” is a hedge word, indicating capability, booster (curved line) “uncover” highlights the theoretical part of the research process, and the attitude marker (bolded) “important” strengthens the value of the study. The following texts used a similar tagging.

*Example 1: The inclusion of theoretically derived, detailed intervening processes can* uncover ***important** pathways of influence. HCR (2021) 47 (2) 192–214*

As noted in Table 2, the size of the corpora in the 1980–1981 period was smaller, to make a comparable diachronic investigation of hyping stance markers over the two periods, the frequencies of the markers were normalized by 1,000 words, a common practice in diachronic discourse studies (i.e., Wu and Pan, 2023). Additionally, the Z-test was calculated to facilitate further statistical understanding of the diachronic changes of the hyping stance markers. Moreover, a *post hoc* qualitative analysis of the most common stance markers of each category and the salient distributional pattern was also conducted to contextualize quantitative findings and further explore how stance markers realized the promotional functions.

## 4 Results

### 4.1 Changing patterns of promotional steps in Discussion sections (RQ1)

The results obtained revealed changes in rhetorical variation in the number and frequency of the promotional steps used in the 1980–1981 and 2020–2021 periods. Table 3 showed an upward trend in both the number of RAs containing the steps, and the total number of the steps in the corpus.

#### 4.1.1 M2S3 highlighting results

M2S3 Highlighting results is categorized as a direct promotional step, which emphasizes key research findings to draw attention to the most significant and noteworthy results of the study. Over the two periods, the number of RAs containing M2S3 increased significantly (*p* = 0.002; *χ*^2^ = 10.026) from just 17 in 1980–1981 to 32 in 2020–2021. The corpus as a whole, however nearly doubled in total steps (from 27 to 61).

Given the longer Discussion sections in the 2020–2021 period, the relative share of M2S3 among all promotional steps rose only by 126%, representing a modest proportional growth despite statistical significance. In other words, while more authors now include this step, its relative prominence within the Discussion section has increased only slightly, suggesting that Communication scholars today are less inclined to overtly foreground their findings, favoring more implicit forms of presentation.

#### 4.1.2 M4S1 pointing out limitations of the current study

M4S1 Pointing out negative features or limitations of the current study is an indirect promotional step that aims to reduce the significance of the contribution and play down the assertiveness of the statement (Lindeberg, 2004; Moreno, 2021). Similar to M2S3, there has been a significant increase in the number of RAs incorporating this step (*p* = 0.019; *χ*^2^ = 6.765), from 16 (40%) to 30 (75%) over the period. The limitation step in the corpus has quadrupled compared to the earlier period, representing the most substantial change among all promotional steps (324%).

When comparing the step frequency with the literature, it was 43% among 7 disciplines (Peacock, 2002), 59% in Empirical Law (Tessuto, 2015), and between 88 and 96% in Applied Linguistics (Montgomery, 2023). Although the limitation step identified in Montgomery’s (2023) study was from both the Discussion and Conclusion sections, the overall changes of the limitation step in the Communication Discussion section align with the literature. Specifically, it suggests that the frequency of discussing limitations was relatively lower in the earlier period but has gradually increased in more recent times.

In other words, there appears to be a general trend for researcher of various disciplines to acknowledge the limitations of their studies more openly. The increase in the use of the limitation step over time may reflect a growing preference for indirect promotional strategies that emphasize caution and transparency, showcasing a more nuanced and self-reflective approach to presenting research findings.

#### 4.1.3 M4S2 stating the contribution or positive features of the current study

M4S2 Stating the contribution or positive features of the current study is categorized as a direction promotion strategy named “boost” in Lindeberg’s (2004) study. As Table 3 indicates, M4S2 is also a significantly increased step (*p* = 0.000; *χ*^2^ = 14.587), the RAs containing this step increased from 19 to 35, marking a fourfold increase in the total steps in the corpus to the earlier period, reflecting a significant change of 249% over time.

Compared to some previous studies, the contribution function was found in 90% of Peacock’s (2002) dataset, 59% in Empirical Law (Tessuto, 2015), and 90% in mixed social science disciplines (Moreno, 2021). These previous studies presented some challenges when trying to align their findings with the present study. Peacock’s study, for instance, categorized other rhetorical steps, such as “recommendations for action,” under the contribution function, while Moreno incorporated both Discussion and Conclusion sections in the dataset.

Despite disciplinary and dataset divergence, different results of these studies do not undermine the importance of this explicit promotional step in closing sections of RAs. Whether in a standalone Discussion (Tessuto, 2015), or combined Results and Discussion sections (Ye, 2019), the contribution step remains a critical element of the research narrative. The diachronic changing pattern of contribution step further signals an evolving trend in Communication RAs, where researchers are increasingly proactive in promoting the significance of their findings.

#### 4.1.4 M4S3 noting specific gaps in other research or practice

M4S3 Noting specific gaps in other research or practice is another direct promotional rhetorical strategy that clearly highlights the value of the authors’ research by pointing out the deficiencies and shortcomings of previous studies (Lindeberg, 2004; Moreno, 2021; Cheng, 2021). Over the two periods, the number of RAs containing the gap step always accounted for the smallest number, but increased significantly (*p* = 0.022; *χ*^2^ = 5.208) from 11 to 21 recently. A similar trend can be found in the number of steps in the corpus (Table 3). These findings align with prior research, which found that the gap step was employed in only 40% of RAs within social science disciplines (Moreno, 2021).

Despite the increase, the relatively infrequent use of the gap step in this study, as well as in others, suggests that the explicit promotional strategy of identifying gaps is still not as commonly employed across the two periods. This may be due to the face-threatening nature of this rhetorical move. Pointing out gaps often involves challenging or refuting prior research, which can be seen as casting doubt on established findings and potentially undermining the perceived credibility of both the researcher and the research community (Myers, 1989; Lindeberg, 2004). The term ‘face-threatening’ is used in a disciplinary rather than interpersonal sense (Myers, 1989), referring to the risk of challenging communal consensus or established research territory, rather than to personal criticism.

#### 4.1.5 M5S1 suggesting the practical applicability of results

M5S1 Suggesting the practical applicability of results is a direct promotional strategy that highlights the real-world practical implications of the research for individuals, organizations, industry, and society. The RAs containing this step are relatively lower, just 11 articles in 1980–1981, but it has significantly increased (*p* = 0.000; *χ*^2^ = 12.832) to 27 in 2020–2021, and a similar increasing trend can be found in steps in the corpus (Table 3).

Compared with the literature, the applicability step was found with an appearance of 70% in Dentistry (Basturkmen, 2012), 69% in Empirical Law (Tessuto, 2015), and 81.7% in Forestry RAs (Joseph and Lim, 2018). It is particularly notable that these abovementioned fields share a strong applied orientation, suggesting that the applicability step may be more prevalent in disciplines where research findings have direct, real-world implications. Given the practical orientation of the Communication study (Craig, 2018), it would be reasonable to expect the increasing prominence of the applicability step in the Communication Discussion section. This explicit promotion strategy would highlight the relevance of findings not only to academic audiences but also to professional practitioners, reflecting the field’s focus on real-world communication issues.

#### 4.1.6 M5S2 making recommendations for future research

M5S2 Making recommendations for future research is also classified as an indirect promotional step that demonstrates to what extent and in which aspect the current contribution inspires direction for future research (Lindeberg, 2004; Moreno, 2021). Of the total six promotional strategies, the recommendation step always accounted for the largest number of RAs containing this step (26 in 1980–1981 and 36 in 2020–2021) that also significantly increased over the periods (*p* = 0.007; *χ*^2^ = 7.168). Similarly, the number of steps in the corpus always constituted the largest amount (Table 3).

Compared with the literature, the recommendation step appears in 59% of RAs (Peacock, 2002), 43% in Empirical Law (Tessuto, 2015), and 79% in qualitative and 94% in quantitative Applied Linguistic RAs (Montgomery, 2023). Similar to the limitation and contribution steps, the recommendation step was also cross-sectionally observed in both the Discussion and Conclusion sections (Tessuto, 2015; Montgomery, 2023). The diachronic increase in recommendation steps in Communication Discussion sections is consistent with the literature, reflecting a growing emphasis among Communication scholars on the continuity and development of the field, similar to trends observed in other disciplines. Additionally, it is notable that the two indirect promotional strategies—limitation and recommendation—accounted for the largest number of steps across both periods. These strategies appear to be interrelated, each playing a complementary role in positioning both the author and their work within the academic community (Yoon, 2017; Montgomery, 2023). By acknowledging the limitations of their studies and simultaneously proposing future research directions, authors effectively frame their contributions as part of an ongoing scholarly conversation, which underscores the dynamic and evolving nature of academic discourse in Communication.

### 4.2 Changing patterns of stance marker (RQ2)

This subsection examines the changing patterns of three hyping stance markers: attitude markers, boosters, and hedges over the period 1980–1981 and 2020–2021. As illustrated in Table 5, there is a substantial surge in all three stance markers, even after normalization by 1,000 words. This finding is in contrast with another diachronic study which the hedge markers, boosters, and attitude markers in the Discussion section of Applied Linguistics during the 1996–2016 period show an overall decreasing trend (Rezaei et al., 2021). Although both studies focus on the Discussion section, the differing patterns of change suggest that stance choices are influenced by disciplinary conventions, temporal contexts, and communicative purposes, even within the same section.

**Table 5:** Change patterns of stance markers.

Stance markers	1980–1981		2020–2021			
	Raw	Normalized	Raw	Normalized	Z-test (*p*-value)	Changes of normalized figure
Attitude markers	78	29.77	217	37.32	0.08	25%
Boosters	95	36.25	336	57.78	0.0000	59%
Hedges	146	55.71	469	80.65	0.0000	45%
Words total	26,205		58,153			

The *p*-value was calculated based on the normalized frequencies of the two periods.

However, we should be cautious in interpreting the change in attitude markers, as the statistical result (*p* = 0.08) did not reach significance at the 0.05 level. In contrast, hedges and boosters, which convey the epistemic aspects of stance such as certainty and possibility, showed a statistically significant change. Attitude markers used to signal the writer’s feelings and affect such as important or interesting, exhibited some degree of variation in promotional rhetoric between the two periods, though this difference was not statistically significant.

In terms of distribution, the hedge markers always comprise the largest amount of the three categories, followed by boosters and attitude markers. This distribution pattern is consistent with stance markers studies in the Applied Linguistics Discussion section (Rezaei et al., 2021; Arizavi et al., 2023; Xie et al., 2024). In other words, although our study only focused on the promotional steps, it reveals a similar overall pattern of stance marker usage across the Discussion section and disciplines over the periods.

In Table 6, the stance markers in all the promotional rhetoric have increased while the preference for the types of stance markers in each step remained unchanged over the two periods.

**Table 6:** Changing pattern of stance markers in promotional steps.

Promotional steps	1980–1981			2020–2021		
	Attitude marker	Booster	Hedge	Attitude marker	Booster	Hedge
M2S3 highlighting	23	15	5	48	44	6
*M4S1 limitation*	13	9	27	31	17	117
M4S2 contribution	21	27	15	69	121	27
M4S3 gap	4	1	8	15	5	28
M5S1 application	5	5	32	21	58	102
*M5S2 recommendation*	12	38	59	33	91	189
Total	78	95	146	217	336	469

The italic steps are indirect promotional steps.

In M2S3, attitude markers made up the highest proportion of hyping stance markers, followed by boosters and hedges. The first two categories exceeded a twofold increase, whereas the rise in hedge markers was only slight. Given that the number of highlighting steps increased by 126% (Table 3), this modest increase observed may actually indicate a declining tendency to use hedges in this step. A similar pattern is observed in M4S2, although boosters accounted for the largest share and showed the most substantial increase (over a fourfold rise), with only a minimal rise in hedges. The shared preference for fewer hedge markers in both steps may be attributed to their communicative purpose that to promote the value and novelty of the research. Since hedges often signal uncertainty, their use could weaken the strength and assertiveness of the claims. A comparable linguistic strategy was observed in the way authors stated research goals or presented findings in the Introduction sections, where self-mention was never combined with hedges (Wang and Zeng, 2021). The authors argued that this avoidance may reflect a concern that hedging could signal an unclear research focus or a lack of confidence in the results. This suggests a broader rhetorical tendency to avoid weakening promotional statements when referring to their own results across sections.

The use of stance markers in the M4S1 limitation and M4S3 gap showed a similar pattern over the two periods: hedge markers were the most frequently used, followed by attitude markers and boosters (Table 6). Although M4S1 focuses on limitations of the current study and M4S3 addresses gaps in previous research, the similar use of stance markers can be attributed to their shared communicative purpose—highlighting research shortcomings and flaws. To achieve these communicative purposes, writers often rely on negative attitude markers such as “neglected” and “question” (for more examples, see Section 4.3.3). However, hedge markers composed the largest amount. This may be because hedge markers function to moderate the writer’s epistemic commitment to a proposition (Xie et al., 2024) and act as a negotiation strategy, signaling writer’s caution when casting doubts on other scholars (Cheng and Unsworth, 2016).

Across the two periods, both the M5S1 application and M5S2 recommendation demonstrated a preference for hedge markers, followed by boosters and attitude markers (Table 6). This preference aligns with that observed in the previous two steps (M4S1 and M4S3), where hedges are used to convey a negotiable tone and maintain flexibility when offering practical and academic suggestions. However, when giving suggestions, writers tend to use boosters rather than attitude markers. When boosters are used in the promotional step, it often expresses an author’s strong conviction and epistemic commitment to assert claims and shut down alternative voices, for example, “demonstrate” and “clearly” (Hyland and Jiang, 2024; also see Section 4.3.4 for more examples). In this way, boosters help maintain an objective and authoritative tone without explicitly conveying personal judgment or emotion, while still promoting the proposed solution.

### 4.3 Stance markers in promotional steps (RQ3)

This subsection examines the changes of stance markers in realization of promotional step between the periods 1980–1981 and 2020–2021.

#### 4.3.1 Hyping stance markers in M3S2 highlighting results

Over the two periods, the positive attitude markers contributed similarly to highlight the results, with boosters intensifying the impact of the value in the studies over the two periods. In Example 2 and 4, the significance of the finding is conveyed by the attitude markers “interesting” and “noteworthy” respectively. In Example 3 and 5, the word “important” and “robust” connotates a positive stance to the finding, further heightened by the inclusion of boosters, “the most.”

*Example 2: Findings related to the research questions examining members’ perceptions of status differentiation within interacting brainstorming groups also suggest some **interesting** conclusions. HCR (1981) 7 (3) 245-258*

*Example 3: From an applied perspective, the most **important** finding to emerge from this study is that the narrative style of news stories employed by newsmagazines tends to…JOACR (1981) 9 (2) 109-119*

*Example 4: Second, our focus on the relationship between content sharing and granular, individual-level political and social factors is **noteworthy**. HCR (2020) 46 (4) 357-384*

*Example 5: We contend that the most **robust** finding from this study pertains to the interplay between individual and community-level resilience and constraints. JOACR (2020) 48 (2) 207–22*

#### 4.3.2 Hyping stance markers in M4S1 pointing out limitations of the current study

In M4S1, the use of hyping stance markers showed a similar quantitative trend, however, authors from the two periods tended to adopt different tones and combinations of these markers. In the 1980–1981 period, the limitation of the studies is expressed in a stronger stance. As illustrated in Example 6, the shortcoming of the study is “far from ideal” and this feeling is intensified by hedging “must” and booster “reiterated.” In Example 7, the word “distasteful” conveys emotionally unpleasant and even offensive while such words category rarely found in the 2020–2021 corpus. In the 2020–2021 period, most limitations function similarly achieved by pointing out deficiencies, but negative attitudes have been mitigated by hedge words, alleviating the negative consequences (Example 8). Most limitations in this period are realized by hedge words (Example 9 and 10) that indicate a cautious attitude due to some uncontrollable factors rather than real faults of the current study.

*Example 6: First, it must be reiterated that the measures of advertising exposure used here are **far from ideal.** HCR (1981) 7 (4) 347-360*

*Example 7: Ethically, it is **distasteful** for many subjects to discuss an immediate tragedy of this magnitude so coldly and clinically. JOACR (1980) 8 (2) 156-160*

*Example 8: However, there may be more specific privacy violations (or subtler privacy invasions) we **neglected** in this study. HCR (2021) 47 (1) 49–74*

*Example 9: This study included a single, relatively small sample of adolescents from the southeastern U.S. – which may limit the generalizability of result. CQ (2021) 69 (5) 525-543s*

*Example 10: Although we have controlled the main features such as intonation and speech rate, other factors may have impacted the results. CR (2021) Dec-15*

#### 4.3.3 Hyping stance markers in M4S2 stating the contribution or positive features of the current study

The communicative function of M4S2 was supported by similar types of hyping stance markers across both periods. However, in 1980–1981, it relied equally on boosters and attitude markers whereas in 2020–2021, the use of boosters nearly doubled that of attitude markers. In the earlier period, the word “important” in Example 11 conveys the significance of the study to real-world implications. In Example 12, the contribution is expressed through the positive attitude marker “value,” with the booster “show” presenting claims categorically as truth (Hyland, 2005). In contrast, the 2020–2021 period demonstrates a heavier reliance on boosters to fulfill the communicative purpose of M4S2. For instance, the adjective “unique” in Example 13 asserts the distinctiveness of the contribution. In Example 14 the contribution and positive features of the studies are evidenced with extensive specific content, while boosters “help” and “unpack” in Example 14 enhance the force of contribution.

*Example 11: …the finding itself has **important** implications HCR (1981) 7 (3) 245–258*

*Example 12: In conclusion, this study shows the **value** of merging interests in interpersonal and mediated communication contexts CR (1981) 16 (1) 59–77 (1)*

*Example 13: Moreover, this study makes a **unique** contribution to the existing body of CIT literature by integrating …CR (2021) June-4*

*Example 14: We help unpack this process by demonstrating the role of emotions in driving OPE for both regime opponents and supporters alike CR (2020) Aug-19*

#### 4.3.4 Hyping stance markers in M4S3 noting specific gaps in other research or practice

The use of hyping stance markers in M4S3 was similar to that in M4S1, with a stronger stance and tone observed in the earlier period. In the 1980–1981 period, the gaps in earlier research are asserted with a stronger stance, evident in phrases such as characterizing prior studies as “dramatizing” in Example 15 and describing them as “not clear” in Example 16. While stance markers implying gaps in the 2020–2021 period are more implicit, “remained a question” in Example 17 signifies past uncertainties, suggesting the potential for resolution at present. While “unfortunately” in Example 18 expresses unfavorable circumstances, but hedge word “few” provides a rationale for the adverse condition, enhancing the acceptability of the gap. It is noteworthy that a large part of the gap steps in 2020–2021 is conveyed through a pattern of negation + modal adverbial hedge, as illustrated in Example 19. In this way, writers avoid confrontation with others’ work, and limit the full illocutionary force of evaluation, toning it down for the writer to express reservation about the proposition (Jiang and Hyland, 2022).

*Example 15: The previous research **dramatizing** the debilitative effects of high CA may, in fact, be realistically interpreted as also illustrating the facilitative effects of low CA relative to the norm of moderate CA. HCR (1980) 6 (2) 146-152*

*Example 16: However, it is **not clear** whether these correlations indicate a “flow “of information, “opinion leadership”, “reinforcement “or other types of social motivations. DCR (1980) 7 (2) 139-160*

*Example 17: Previous meta-analyses have shown that narratives can persuade (Braddock and Dillard, 2016; Shen et al., 2015; Tukachinsky and Tokunaga, 2013), but whether it is through reducing resistance **remained a question**. HCR (2020) 46 (4) 412-443*

*Example 18: **Unfortunately,** few studies have investigated sexpositive constructs that emphasize the normativity of sexuality. CQ (2021) 69 (5) 525-543*

*Example 19: These types of online communities have not been explored widely due to access limitations. CQ (2021) 69 (5) 501-524*

#### 4.3.5 Hyping stance markers in M5S1 suggesting the practical applicability of results

In M5S1, unlike other promotional steps, the attitude polarity, level of force, and tentativeness, which highlight the implications of the results, remained largely stable across the two periods. In both periods, M5S1 conveys the value of the findings through positive attitude markers, such as “worthwhile” and “important” in Examples 20 and 22. It is noteworthy that this emphasized value is strategically mitigated by the hedge word “may” in both examples. In most cases, when offering practical suggestions, the word “should” is used frequently (Examples 21 and 23), indicating a higher level of confidence in the statements. However, the suggestions made in the recent period (Examples 23) are presented through a mixed use of hedges and boosters, reflecting a more delicate and cautious manipulation of a persuasive tone in proposing practical implications.

*Example 20: Although there is often a tendency to concentrate on production variables to enhance learning, it also may be **worthwhile** to try to change or develop perceptions. CR (1980) 7 (1) 121–135*

*Example 21: In future studies, students should try doing related activities during the last few sessions, such as speaking into a tape recorder, reading from a book, answering questions, or giving impromptu speeches. CQ (1980) 28 (4) 47–56*

*Example 22: it is **important** to increase a person’s health consciousness in order to trigger autonomous motivation, which may sustain long-term behavioral changes for obesity prevention. JOACR (2021) 49 (2) 228–245*

*Example 23: We should also note that our findings are mostly applicable to recent examples of right-wing populists’ critique on the legitimacy of the established order. CR (2020) Oct-20 (1)*

#### 4.3.6 Hyping stance markers in M5S2 making recommendations for future research

In both periods, M5S2 underscores the research value by linking current findings to future studies, though authors employed different combinations of hyping stance markers. Over the periods, the hedge word “should” that aims to improve the force of suggestion is used frequently in M5S2 (Example 24 and 27). Boosters are frequently employed to convey a strong sense of certainty regarding future research directions (Example 25 and 26), reflecting a strong ambition to continue the current line of research. Similar to M5S1, in the 2020–2021 period, the research recommendation is achieved through some subtle use of stance markers. As indicated in Example 26, the voice of recommendation is strong but in a positive collaboration manner through booster “insights.” While hedge words “could” and “would” in Example 26 suggest the possibility and interpretative aspect of a claim, enhancing the acceptability of the recommendation, and also a cautious manner when giving suggestions.

*Example 24: Future research should focus on the effect of viewing contradictory models. HCR (1980) 6(4) 340–351*

*Example 25: Certainly, additional research is needed to examine what cues are leaked during emotional concealment. HCR (1981) 7 (4) 325–339*

*Example 26: Finally, conducting an experimental study could reveal causal relationships between variables that would offer additional insights beyond what survey research can provide. JOACR (2020) 48 (6) 695–713*

*Example 27: Future studies should examine more closely the cognitive aspects of credibility evaluations using psychological methods CR (2020) APR-4*

## 5 Discussion

This study investigated the changes of 6 promotional steps and 3 types of hyping stance markers in Communication Discussion sections over the period 1980–1981 and 2020–2021. Over the two periods, all types of promotional steps and hyping stance markers have increased even after normalization, showing a distinctive pattern of promotion in Communication Discussions. The following discussion interprets these patterns through an inductive lens, highlighting rhetorical tendencies that emerged from the corpus.

Over the two periods, both the number of RAs containing promotional steps and the total number of these steps in the corpus show an upward trend. This finding is consistent with observations in Medical RA abstracts, where persuasive rhetorical steps such as claiming the importance of the research topic, indicating gaps, and stating the contribution have increased significantly since the 1990s (Martín and León Pérez, 2024).

Among the overall increase, the rise of the two indirect promotional steps, M4S1 limitation, and M5S2 recommendation is particularly notable. Over the period, M4S1 showed the largest proportional increase, while M5S2 consistently remained the most frequently employed rhetorical strategy. The preference for the two indirect promotional strategies has been also observed in recent Applied Linguistics corpora (Montgomery, 2023; Deng et al., 2024). Another noticeable increase is seen in M5S1 applicability, with the number of Discussion sections containing this step nearly tripling over the periods. The significant increase M5S1 may be attributed to the inherently practical nature of the discipline. As Craig (1999) argued, Communication studies aim to cultivate the art of communication through critical inquiry, positioning the field as a practical discipline that reflects on real-world problems, technologies, and principles. This practical orientation likely motivates scholars to offer actionable recommendations in their Discussions, as a way to bridge theory and practice and contribute to the improvement of real-world communication. This preference for limitation, recommendation, and applicability steps suggests a tendency toward implicit forms of promotion, where persuasive intent is realized through caution, practicality, and reflexivity rather than overt self-assertion.

There is a substantial surge in all three hyping stance markers, even after normalization. However, the changing pattern observed in our study diverges from previous literature, where the use of hedges, boosters, and attitude markers in the Discussion sections of Applied Linguistics articles showed a decreasing trend (Rezaei et al., 2021), or fluctuations in the case of boosters (Xie et al., 2024). Both studies suggest a shift toward a more cautious rhetorical stance, likely in response to the growing influence of “scientism” and the conventions of objective writing in the social sciences.

Nevertheless, we are cautious about concluding that the promotional tone in the Communication Discussion sections deviates from the norms of scientism or objective scholarly discourse in social science disciplines. Rather, our findings suggest that persuasion in these sections tends to occur in a more subtle and restrained manner. This tendency can be attributed to the study’s specific focus on promotional steps. While overt stance strategies such as attitude markers exhibited only a statistically insignificant increase, there was extensive use of hedging devices. These elements served to temper the strength of claims, manage authorial positioning, and enhance the perceived credibility of the argument in a subtle and deliberate way.

Our qualitative analysis of hyping stance marker distribution revealed that, although the overall pattern remained stable, writers increasingly employed more strategic combinations of markers when highlighting research findings and contributions, making suggestions, and identifying research limitations. This deliberate use of rhetorical strategies accounts for the increased frequency of hyping stance markers observed in the 2020–2021 corpus. When used together, these markers helped refine the tone and strengthen the persuasive impact of the authors’ claims in the Discussion sections.

Although no directly comparable studies have examined the distribution of hyping stance markers across specific promotional steps, existing research reveals a similar tendency in the combined Engineering Results and Discussion sections to strategically combine boosters and hedges (Lee and Casal, 2014). They argued that this combination allows authors to present their claims with both caution and assertiveness, thereby cultivating a relationship with readers that, while not necessarily persuasive in a definitive sense, maintains an inherently influential tone. A similar strategy is also observed in the management of academic conflict in Applied Linguistics Discussion sections, where interpersonal resources “entertain” (e.g., may, suggest) counted the highest frequency (Cheng and Unsworth, 2016). They argued that this rhetorical choice implicitly acknowledges the validity of competing viewpoints within a heteroglossic context and helped mitigate the risk of readers explicitly rejecting the writer’s argument.

Our findings that the Communication Discussion sections are becoming more promotional, as reflected in the increasing number of promotional steps and stance markers, align with previous diachronic studies in Medical Science (Wen and Lei, 2022; Cao et al., 2021; Martín and León Pérez, 2024), Science (Yuan and Yao, 2022), and Health Grant Applications (Millar et al., 2022). However, promotion in the Communication Discussion sections tends to be implicit, subtle, and strategic, a pattern that is aligns more closely with the discourse convention of other social science fields, where persuasion operates through balance, reflexivity, and rhetorical control rather than through overt self-promotion.

In cross-disciplinary diachronic studies, the use of attitude markers declined in soft disciplines but increased in hard disciplines between 1985 and 2015 (Hyland and Jiang, 2016). Positive words also appeared more frequently in Abstracts of hard disciplines than in those of soft disciplines (Liu and Zhu, 2023), and the increase in positive language in Political Science was far less dramatic (4-fold) compared to Medical Science (10-fold) over a similar period (Weidmann et al., 2018). In a study of promotional rhetoric in Introduction sections, the step of stating the value of the present research has also been observed with greater frequency in Health Science RAs than in other fields (Martín and León Pérez, 2014).

Scholars have attributed the growing use of promotional rhetoric in science disciplines to mounting competition for academic positions, tenure, research visibility, and funding (Liu and Zhu, 2023; Caulfield, 2018; Bordignon et al., 2021; Edlinger et al., 2023). However, the research environment does not fully support the assumption. In fact, competition in the social sciences can be even more intense than in the natural sciences. In recent years, university departments in the humanities and social sciences have been increasingly eliminated in countries such as Japan (Jenkins, 2015) and China (Wang, 2025). Similar cutbacks in courses and programs have been observed at Harvard University (Parker and Sykes, 2024), as well as at the Universities of Sheffield, Chester, and Cumbria in the UK (Lee, 2021). Funding for social science research has been significantly reduced, including cuts by the U.S. Department of Defense (Kupferschmidt, 2025) and major reductions to New Zealand’s Marsden Fund (Gordon, 2024). The research climate may impose even greater pressure on social scientists to justify the value and contribution of their work, leading to the adoption of increasingly promotional and persuasive rhetorical strategies. However, the findings of this study, together with others, suggest that promotion strategies are often employed in more implicit and nuanced ways in social science disciplines.

Another noteworthy outlook toward genre is its rhetorical intent, and it is being a form of social action that constructs meaning through the mediation between private intentions and socially recognized needs (Miller, 1984). The cautious tone commonly found in humanities and social science writing may reflect intellectual humility, but it can also be seen as a strategic response to the marginalization of these fields in a research culture increasingly shaped by scientism (Cobern and Loving, 2001; Honeycutt and Jussim, 2023; May, 2021). This culture, which prioritizes empirical certainty, technical precision, and measurable outcomes, has become increasingly influential even within the social sciences themselves (Halford and Savage, 2017). Although a full exploration of the long-term effects of this trend lies beyond the scope of this paper, it prompts a pressing question: might interpretive, critical, and value-oriented forms of inquiry be gradually supplanted by technocratic and scientistic models of knowledge production?

## 6 Conclusion

By examining six promotional steps and three types of hyping stance markers in Communication Discussion sections across two time periods (1980–1981 and 2020–2021), this study identified a notable increase in promotional features, particularly those indirect strategies. Both strategies have increased and are often employed in implicit, subtle, and strategically calibrated ways. These patterns reflect a broader trend in the social sciences toward intellectual humility, a rhetorical adaptation to an increasingly scientistic research culture that privileges objectivity and measurable outcomes. As discussed in Section 2.3, objectivity in academic writing is not the absence of stance but a rhetorical performance of impartiality and evidential reasoning. Such discursive practices enable writers to project epistemic credibility while minimizing overt self-promotion. The rise of subtle promotional strategies observed in this study therefore illustrates how contemporary authors negotiate the tension between persuasion and objectivity, constructing credibility through restraint rather than overt assertion.

### 6.1 Implication

The increasingly implicit, subtle, and strategically calibrated forms of promotion observed in the 2020–2021 corpus reveal how authors navigate the publish or perish culture that rewards confidence and contribution. Yet this culture has also been widely criticized for distorting science, catering to the attention economy, and fostering academic capitalism (Millar et al., 2019; West and Bergstrom, 2021; Hyland, 2023). Against this backdrop, rhetorical modesty and reflexive transparency, evident in the growing use of limitation and recommendation steps and in more measured and balanced expressions of research significance, should not be seen as signs of weakness but as ethically responsible ways of communicating contribution. For editors and reviewers, the traditional assumption that disclosing limitations undermines a paper’s credibility should be reconsidered (Brutus et al., 2013). Instead, editorial and review policies should encourage authors to discuss limitations and future directions openly, without fear of negative evaluation. Strategically modest forms of promotion that balance visibility with integrity ought to be valued as markers of scholarly maturity and credibility. Fostering this balance may help realign research communication with its epistemic and social purposes.

### 6.2 Limitation and recommendation for future research

The limitations identified in this study highlight areas that warrant additional exploration. Firstly, the findings of this study are based on Communication Discussion section, which may not be fully generalizable to other sections or disciplines. Future research could explore similar diachronic changes in the Introduction sections of other disciplines, which, as the mirror image of the Discussion section (Swales, 1990), tend to exhibit a symmetrical relationship in terms of promotional moves (Deng et al., 2024) and often contain a higher frequency of hyping language (Millar et al., 2019). Secondly, our study focused solely on the distribution of hyping stance markers within promotional steps. However, non-promotional steps such as M3S2 Comparing with previous research, may also feature hedge markers, particularly when addressing research conflicts. While our approach does not capture the full range of hyping stance marker usage across all rhetorical steps, it provides a comprehensive understanding of how these markers function in the realization of promotional strategies. Thirdly, despite the precautions taken to ensure comparability, a degree of asymmetry remains between the two sub-corpora. As a common limitation in diachronic research, this issue was mitigated by including the same number of Discussion sections in each period and normalizing the counts of hyping stance markers. Future studies could extend this work by incorporating a larger and more balanced corpus, particularly from more recent years.

## Data Availability

The datasets presented in this study can be found in online repositories. The names of the repository/repositories and accession number(s) can be found at: https://www.iris-database.org/details/Yzk3N-qKRsg.

## Footnotes

1 The authors gratefully acknowledge the anonymous reviewers for their helpful suggestion to clarify the conceptual distinction between “promotion” and “hype.”

## AppendixAppendix A Change of words and macrostructure over the two-periods.

**Table:**

	CDS-1	CDS-2	% Changes
No. of RAs	177	212	20%
RAs with D	96 (54%)	152 (71%)	31%
Selected No.	40	40	
% to total RAs^4.2^	23% (40/177)	19% (40/212)	−17%
Words total	26,205	58,153	122%
